# Ischaemic stroke and the recanalization drug tissue plasminogen activator interfere with antibacterial phagocyte function

**DOI:** 10.1186/s12974-017-0914-6

**Published:** 2017-07-21

**Authors:** Antje Vogelgesang, Claudia Lange, Lara Blümke, Georg Laage, Sarah Rümpel, Sönke Langner, Barbara M. Bröker, Alexander Dressel, Johanna Ruhnau

**Affiliations:** 1 Department of Neurology, University Medicine Greifswald, Fleischmannstraße 41, FC3, 17475 Greifswald, Germany; 2 Department of Immunology, University Medicine Greifswald, Greifswald, Germany; 3 Department of Diagnostic Radiology and Neuroradiology, University Medicine Greifswald, Greifswald, Germany; 4 Department of Neurology, Carl-Thiem Klinikum, Cottbus, Germany

**Keywords:** Stroke, r-tPA, NETosis, Oxidative burst, Phagocytosis, Innate immune response, Hormones

## Abstract

**Background:**

Stroke induces immune alterations such as impaired oxidative burst and reduced release of neutrophil extracellular traps (NETs). We hypothesised that key enzymes of these defence mechanisms may be altered in ischaemic stroke. Therefore, we analysed the intra- and extracellular amounts of myeloperoxidase (MPO) and neutrophil elastase (NE) in patient sera and granulocytes and monocytes. Because the autonomous nervous system is thought to mediate stroke-induced immune alterations, we also studied the influence of stress hormones and acetylcholine on MPO and NE.

Rapid recanalization by recombinant tissue plasminogen activator (r-tPA) is the only available treatment for ischaemic stroke besides thrombectomy, and its influence on antibacterial defence mechanisms of granulocytes and monocytes were addressed here.

**Methods:**

Ex vivo*:* Intracellular and serum MPO and NE were measured on days 0, 1, 3 and 5 post-stroke by either flow cytometry or enzyme-linked immunosorbent assay (ELISA) and compared to controls. In vitro*:* Blood from healthy donors was incubated with catecholamines, dexamethasone and acetylcholine, and the percentage of NET-producing cells and the area covered by NETs were quantified immunohistochemically. Intra- and extracellular MPO and NE were quantified by flow cytometry or ELISA. Blood samples from healthy donors were incubated with r-tPA, and oxidative burst, phagocytosis, NETosis, cytokine release, MPO and NE were quantified by flow cytometry, ELISA and microscopy.

**Results:**

MPO was reduced in granulocytes but increased in sera obtained from stroke patients compared to controls. NE was not altered intracellularly but was elevated in patient sera. The percentage of NET-producing neutrophils was decreased by stress hormones and increased by acetylcholine. Neither intracellular MPO nor NE was altered by hormone treatment; however, adrenaline and acetylcholine induced NE release.

r-tPA led to reduced phagocytosis and oxidative burst in granulocytes and monocytes in vitro. NETosis, MPO release and cytokines were not altered, whereas NE release was enhanced by r-tPA.

**Conclusions:**

Intracellular reduction of MPO might be responsible for reduced NETosis in stroke patients. The impact of enhanced MPO and NE serum levels in stroke patients should be addressed in future studies.

r-tPA impaired antibacterial defence function in vitro. Therefore, patients who undergo unsuccessful recanalization therapy might be at higher risk for infection, which should be analysed in future investigations. Immune alterations due to r-tPA effects in stroke patients should also be investigated.

**Electronic supplementary material:**

The online version of this article (doi:10.1186/s12974-017-0914-6) contains supplementary material, which is available to authorized users.

## Background

Stroke induces profound immune alterations, which include the suppression of innate antibacterial defence mechanisms predisposing patients to post-stroke infections [1–3]. Such infections are reported in approximately 30% of stroke patients and are associated with worse outcomes and increased post-stroke mortality [4]. These immune alterations are thought to be initialized by high concentrations of catecholamines and glucocorticoids, which are regularly observed post-stroke in mice and in humans [5–9]. In animal models, inhibition of the sympathetic nervous system by propranolol or interruption of the parasympathetic nervous system by vagotomy reversed lymphocytic dysfunction and lowered the incidence of bacteraemia and pneumonia, whereas blocking the hypothalamus-pituitary gland axis by Ru486 normalised lymphocyte numbers and HLA-DR expression in monocytes [10, 11]. We previously reported that the oxidative burst, a mechanism that produces free oxygen radicals to kill phagocytosed bacteria, is impaired in ischaemic stroke patients in both granulocytes and monocytes. In addition, the amount of neutrophil extracellular traps (NETs) released by neutrophils in post-stroke patients is lower than in healthy subjects [1]. These traps, formed of DNA, prevent bacteria from further spreading and kill bacteria via high local concentrations of enzymes and bactericidal molecules that are attached to the DNA [12].

The enzymes myeloperoxidase (MPO) and neutrophil elastase (NE) are involved in the key steps of oxidative burst and NETosis. MPO produces antibacterial hypochlorous acid. Upon reactive oxygen species (ROS) production, NE escapes the azurophilic granules and translocates to the nucleus. MPO binds to chromatin in later stages, and both enzymes cooperatively enhance chromatin decondensation, leading to cell rupture and NETosis [13].

Recombinant tissue plasminogen activator (r-tPA) is the only available treatment for stroke patients besides mechanical thrombectomy, which both target rapid recanalization of the blocked vessels. The beneficial effect of recanalization by r-tPA is undisputed; however, experimental stroke studies have also reported neurotoxic effects of r-tPA [14, 15]. It was previously reported that r-tPA leads to degranulation of neutrophils, thus increasing NE and MPO in serum [16], and that r-tPA reduces stroke lesion size or outcomes independent of successful recanalization [17, 18]. Potential off-target effects of r-tPA on innate bacterial defence mechanisms of granulocytes and monocytes have not yet been studied; they could, however, be relevant for patient outcomes.

We therefore investigated the influence of (i) ischaemic stroke, (ii) stress hormones and the neurotransmitter acetylcholine and (iii) r-tPA on intra- and extracellular NE and MPO levels in patient blood samples and in cell cultures.

## Methods

### Study aim, design and setting

#### Ex vivo analyses of patient-derived samples

This prospective explorative study recruited patients from the dedicated stroke unit of the University Medicine Greifswald. We investigated the effects of ischaemic stroke on the key enzymes of NETosis and oxidative burst ex vivo. MPO and NE were quantified intracellularly and in sera in peripheral blood samples obtained within 12 h of stroke onset and thereafter on days 1, 3 and 5.

Patients aged ≥18 years suffering from middle cerebral artery (MCA) infarct were eligible for this study within 12 h after the onset of symptoms if their National Institute of Health Stroke Scale Score (NIHSS) was ≥6 and if no signs of systemic infection were detected on admission (C-reactive protein ≤50 mg/L and procalcitonin ≤0.5 ng/mL). Patients receiving immunosuppressive drugs or diagnosed with a malignancy were not recruited. Patients were treated with the best medical care. Recanalization with r-tPA and/or thrombectomy was carried out as clinically indicated. Age-matched control individuals were either healthy volunteers or were recruited from the ophthalmology clinics among patients scheduled to receive cataract surgery. Controls were neurologically and immunologically healthy. See the participants’ characteristics in Tables 1 and 2.

**Table 1:** Subjects characteristics: myeloperoxidase (MPO) extra- and intracellular

		Total no.	Age (years)^a^	NIHSS^b^	Lesion volume (mm^3^)^c^	Male	Female	Thrombolysis	Thrombectomy
Extracellular^f^ (Fig. 1a)	Control subjects	11	79 (69–86)	NA	NA	6	5	NA	NA
	Stroke patients	23	73 (52–92)	9 (6–22)	55.97 (3.69–411.49)^e^	15	8	10	1
Intracellular (Fig. 1b)	Control subjects	14	75 (67-83)	NA	NA	8	6	NA	NA
	Stroke patients	20	75 (52–89)	9 (6–20)	21.61 (2.82–104.15)^d^	10	10	16	0

*NA* not applicable

^a^Mean (range)

^b^Median (range)

^c^Median (range)

^d^11 without demarkated infarct area

^e^8 without demarkated infarct area

^f^Experiments were conducted using the same study population

**Table 2:** Subjects characteristics: neutrophil elastase (NE) extra- and intracellular

		Total No.	Age (years)^a^	NIHSS^b^	Lesion Volume (mm^3^)^c^	Male	Female	Thrombolysis	Thrombectomy
Extracellular^f^ (Fig. 1c)	Control subjects	11	79 (69–86)	NA	NA	6	5	NA	NA
	Stroke patients	23	73 (52–92)	9 (6–22)	55.97 (3.69–411.49)^e^	15	8	10	1
Intracellular (Fig. 1d)	Control subjects	10	73 (63–86)	NA	NA	4	6	NA	NA
	Stroke patients	12	71 (30–85)	18 (8–22)	52.12 (1.67–312.43)^d^	5	7	7	5

*NA* not applicable

^a^Mean (range)

^b^Median (range)

^c^Median (range)

^d^11 without demarkated infarct area

^e^8 without demarkated infarct area

^f^Experiments were conducted using the same study population

#### Determination of stroke lesion size

Routine cerebral computed tomographic (cCT) images (sequential cCT native, 4.5 mm layer thickness supra- and infratentorial; mAs = 50; kV = 120) were acquired on a 16-row multislice CT scanner (Somatom 16, Siemens Medical Systems, Erlangen, Germany). Images were analysed using OSIRIX 5.6. To calculate the infarct size, the regions of interest were defined manually and the lesion volume was calculated semi automatically.

#### In vitro studies

To analyse whether catecholamines, dexamethasone or acetylcholine account for the observed alterations in NETosis post-stroke, blood samples from healthy donors were incubated with the specified hormones and transmitters for 4 h. NETosis was induced, and the NET area and percentage of NET-releasing cells were immunohistochemically quantified. Additionally, intracellular MPO and NE were determined by flow cytometry and ELISA after 24 h of co-culture.

To analyse the influence of r-tPA on antibacterial defence, peripheral blood mononuclear cells (PBMCs) from healthy subjects were incubated with 0, 0.5 or 1 μg/ml r-tPA for 4 h, and oxidative burst, phagocytosis, NETosis, MPO and NE were quantified by flow cytometry, ELISA and microscopy. The release of cytokines was analysed after 72 h of incubation with r-tPA. r-tPA dosage was chosen to mimic the levels that are found in patients.

### Phagocytosis

During phagocytosis, bacteria are engulfed by specialised phagocytes such as monocytes and neutrophils. After labelling cells with anti-CD14 antibody to distinguish monocytes (hCD14 APC; clone M5E2; BD Biosciences), a Phagotest Kit® (Biotechnology GmbH, Heidelberg, Germany) was used according to the manufacturer’s instructions to quantify phagocytosis. Cells were incubated with FITC-labelled opsonized *Escherichia coli.* Engulfment of *E. coli* was stopped by placing the samples on ice after 10 min, and the FITC-signal of non-engulfed *E. coli* was quenched. The percentage of phagocytosing cells and the amount of engulfed *E. coli* per single cell were determined by flow cytometry. The results were evaluated using FlowJo Software 7.6.5 (Tree Star Inc., Ashland, OR, USA).

### Oxidative burst

The intracellular production of free oxygen radicals during oxidative burst is a key bacterial defence mechanism. After labelling cells with anti-CD14 antibody (hCD14 APC; clone M5E2; BD Biosciences) to identify monocytes, the respiratory burst was measured using a Phagoburst Kit® (Glycotope Biotechnology GmbH) according to the manufacturer’s instructions. Cells were left unstimulated or were incubated with *E. coli*, phorbol 12-myristate 13-acetate (PMA), or *N*-formylmethionine-leucyl-phenylalanine (fMLP) as stimulants for 10 min at 37 °C. Conversion of dihydrorhodamine 123 to rhodamine 123 enabled quantification of reactive oxygen species by flow cytometry. The percentage of phagocytes that underwent oxidative burst and the enzymatic activity per cell, given by the mean fluorescence intensity (MFI), were quantified and evaluated using FlowJo Software 7.6.5 (Tree Star Inc., Ashland, OR, USA).

### Quantification of NETosis

To determine the capacity of neutrophils to release NETs, cells were isolated and cultured as described previously [1]. NETosis was induced by incubating either fMLP (455 nmol/l) or PMA (736 nmol/l) for 2 h at 37 °C in 5% CO_2_. Unstimulated neutrophils served as a negative control. DNA was stained with *SYTOX*®-Green Nucleic Acid Stain (Invitrogen, Eugene), and both the total cells and NET-forming cells were quantified by microscopy (LEICA DBMI-4000b). NET-releasing cells were defined by a fluorescent area of ≥300 μm^2^. The percentage of cells releasing NETs and the total area covered by NETs/cell were calculated Additional file 1.

### Intracellular NE and MPO staining

NE and MPO are essential enzymes involved in oxidative burst and NET formation. To measure the intracellular NE and MPO amount, these enzymes where stained for flow cytometric analysis. Whole venous blood anticoagulated with ethylenediaminetetraacetic acid (EDTA) was lysed in accordance with the instructions of Human FoxP3 Buffer Set (BD Biosciences, Heidelberg, Germany). Nonspecific binding was blocked by Fc Receptor Blocking Reagent for human samples (Miltenyi Biotec GmbH, Bergisch Gladbach, Germany). MPO was stained by fluorescein isothiocyanate (FITC)-labelled antibody according to the manufacturer’s instructions for the FITC anti-human flow kit (Biolegend, San Diego, CA, USA). For NE staining, cells were incubated with anti-human-NE as a primary antibody (DAKO, Glostrup, Denmark). Phycoerythrin (PE)-labelled goat anti-mouse immunoglobulin G (minimal-x-reactivity, Biolegend, San Diego, CA, USA) served as a secondary antibody. CD14 surface expression was detected by allophycocyanin (APC)-coupled anti-human CD14 antibody (Biolegend, San Diego, CA, USA). Intracellular amounts of NE and MPO were measured using a BD LSR II flow cytometer (BD Biosciences, San Jose, CA, USA) by determining the MFI. Isotype controls and FMO controls were used as appropriate.

### In vitro hormone and neurotransmitter assays

Transmitters of the sympathetic and parasympathetic nervous systems and cortisol as an effector of the hypothalamic–pituitary–adrenal axis have been shown to induce immune alterations post-stroke. To determine their in vitro effects on NETosis and oxidative burst, whole blood from healthy donors was pre-incubated for 4 h (for NETosis at room temperature; for oxidative burst at 37 °C) in 5% CO_2_ (for NETosis, 0.04% CO_2_) in the presence of either adrenaline (1 × 10^−7^ mol/l; 1 × 10^−5^ mol/l), noradrenaline (1 × 10^−7^ mol/l; 1 × 10^−5^ mol/l), acetylcholine (5.5 × 10^−6^ mol/l; 5.5 × 10^−4^ mol/l) (adrenaline, noradrenaline, acetylcholine from Sigma, Deisenhofen, Germany) or dexamethasone (2.5 × 10^−7^ mol/l; 2.5 × 10^−6^ mol/l) (MerckPharmaGmbH, Darmstadt) followed by neutrophil isolation and induction of NETosis or oxidative burst. In addition, we quantified intracellular and extracellular MPO and NE by flow cytometry and ELISA after 24 h of hormone or acetylcholine exposure. RPMI (supplemented with 2% glutamine, 2% penicillin/streptomycin, 10% AB-Sera, 1% HEPES (4-(2-hydroxyethyl)-1-piperazineethanesulfonic acid) without hormones or acetylcholine served as controls. Hormone concentration was chosen to span the range that is found in patients’ post-stroke.

### In vitro r-tPA assays

r-tPA is the most commonly used treatment for rapid recanalization in the acute phase of cerebral ischaemia. To study its impact on antibacterial immune defence mechanisms, whole blood samples from healthy donors were incubated for 4 h with 0.5 or 1 μg/ml r-tPA or control buffer alone in vitro. The phagocytes were tested for phagocytosis, oxidative burst and NETosis as described above. Intracellular amounts of MPO and NE were examined by flow cytometry.

To determine the effect of r-tPA on cytokine patterns, cells were incubated for 72 h before supernatants were obtained.

### MPO-ELISA and NE-ELISA

Commercially available kits were used to measure MPO and NE in the supernatants of samples incubated with or without r-tPA as described above according to the manufacturer’s instructions (MPO: Biolegend, San Diego, CA, USA; NE: Assaypro, St. Charles, MO, USA).

### Cytokine assay

Venous blood was sampled into (i) EDTA or (ii) heparin vacutainers, and PBMCs were isolated by standard density centrifugation (Biocoll Separating Solution, Biochrom AG, Berlin, Germany). Then, 200,000 cells per well were (i) stimulated with PHA (1 μg/ml) in 96-well round-bottom plates or (ii) placed in anti-CD3/anti-CD28 (purified NA/LE mouse anti-human CD3 and purified NA/LE mouse anti-human CD28, both BD Pharmingen, San Jose, USA) pre-coated 96-well flat-bottom plates and incubated with r-tPA for 72 h. The production of INF-γ, IL-2, IL-4, IL-5, IL-6, IL-9, IL-10, IL-13, IL-17A, IL-17F, IL-21, IL-22 and TNF-α was measured using a LegendplexTM Multi-Analyte Flow Assay Kit (Biolegend, San Diego, CA, USA) in the supernatants of PBMCs according to the manufacturer’s instructions.

### Statistical analyses

All datasets were tested for adherence to a Gaussian distribution via the Kolmogorov–Smirnov test. Multiple comparisons of Gaussian-distributed data were performed using analysis of variance, and the Bonferroni correction for multiple comparisons was used as a post-test. When some of the experimental data failed the normality test, non-parametric testing was used throughout. ANOVA or repeated measures ANOVA, the Kruskal–Wallis test with Dunn’s multiple comparison test as a post-test or the Friedman test were used as appropriate. Post-tests were only performed when initial testing revealed significant differences between groups. Correlations were determined by Spearman analysis. GraphPad-PRISM 5.0 (GraphPad Software Inc., San Diego, CA, USA) was used for all analyses. A *p* value <0.05 was regarded as significant. Gaussian-distributed data are shown as the mean ± SD. Other data are shown as the median [min–max].

## Results

### Intracellular MPO reduction and increased NE and MPO release in stroke patients

To explain the reduced respiratory burst and impaired NETosis after stroke, we analysed the intracellular amounts of MPO and NE in patients’ granulocytes and monocytes and compared the results to those obtained with cells from age- and sex-matched healthy subjects.

**Fig. 1:**
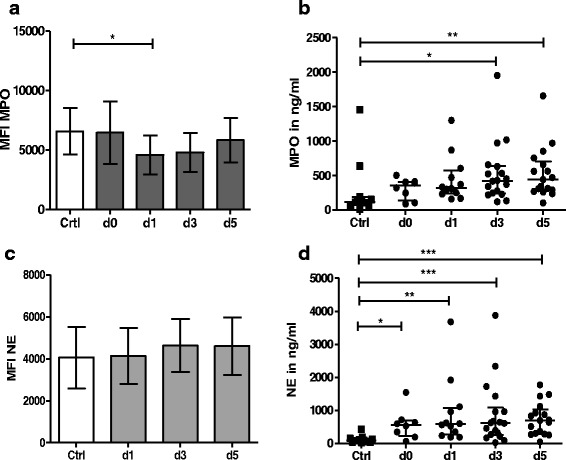
NE and MPO in stroke patients versus controls. Stroke patients were analysed and compared to healthy controls. MPO and NE levels were measured intracellularly in granulocytes by flow cytometry (**a, c**) and are reported as MFI and in the sera of patients using ELISA (**b**, **d**) and reported in ng/ml. MPO was estimated on the day of stroke admission (d0), day 1 (d1), day 3 (d3) and day 5 (d5) by flow cytometry (**a**; *n*_crtl_ = 14, *n*_d0_ = 14, *n*_d1_ = 18, *n*_d3_ = 14, *n*_d5_ = 15). The extracellular amount of MPO was measured by ELISA (d0, d1, d3, d5) (**b**; *n*_crtl_ = 11, *n*_d0_ = 8, *n*_d1_ = 12, n_d3_ = 18, *n*_d5_ = 17). The intracellular amount of NE was measured on the day after stroke (d1) and on days 3 and 5 (d3, d5) (**c**, *n*_crtl_ = 10, *n*_d1_ = 10, *n*_d3_ = 10, *n*_d5_ = 10). NE in the sera was analysed on the day of stroke admission (d0) and on days 1, 3 and 5 after stroke (d1, d3, d5) (**d**; *n*_crtl_ = 11, *n*_d0_ = 8, *n*_d1_ = 12, *n*_d3_ = 18, *n*_d5_ = 17). *p < 0.05; **p < 0.01; ***p < 0.005. Mean and SD ranges for **a** and **c** and medians and interquartile ranges for **b** and **d** are given. ANOVA and Bonferroni comparisons as post hoc tests were used for data in **a** and **c**; data in **b** and **d** were assessed by Kruskal–Wallis test and Dunn’s multiple comparison test

On day 1 post-stroke intracellular MPO was significantly reduced in stroke patients’ granulocytes compared to controls (MPO–MFI_d1(mean ± SD)_: 4598 ± 1642 vs. MPO–MFI_Control_: 6580 ± 1955; *p* = 0.0136) (Fig. 1a). The reduction was transient. The same trend was observed in monocytes (see Additional file 2A). By contrast, MPO serum levels steadily increased during the observation period, and differences with controls became significant from day 3 post-stroke (MPO_d3(median [min-max])_ 422 ng/ml [118–1952 ng/ml], MPO_d5(median [min-max])_ 441 ng/ml [101–1656 ng/ml] vs. MPO_Control(median [min-max])_ 115 ng/ml [13–1453 ng/ml]; *p* = 0.0176 (Fig. 1b).

Intracellular NE amounts were not altered in stroke patients’ granulocytes (Fig. 1c) (NE–MFI_d1(mean ± SD)_ 4136 ± 1343; NE–MFI_d3_ 4639 ± 1259; NE–MFI_d5_ 4610 ± 1368 vs. NE–MFI_Control_ 4055 ± 1473) nor monocytes (NE–MFI_d1(mean ± SD)_ 2457 ± 1002; NE–MFI_d3_ 3027 ± 725; NE–MFI_d5_ 2839 ± 737 vs. NE–MFI_Control_ 3211 ± 1056) (see Additional file 2B). NE in the sera of stroke patients was significantly increased after stroke on all days investigated (NE_Control(median [min–max]_ 98 ng/ml [26–430 ng/ml], NE_d0(median [min–max]_: 565 ng/ml [64–1542 ng/ml], NE_d1(median [min-max]_ 584 ng/ml [188–3678 ng/ml], NE_d3(median [min-max]_ 619 ng/ml [27–3874 ng/ml], NE_d5(median [min–max]_ 697 ng/ml [49–1772 ng/ml]; *p* = 0.0007) (Fig. 1d). None of the intra- und extracellular MPO and NE parameters correlated with stroke severity or neutrophil numbers.

### Hormones and acetylcholine do not influence intracellular MPO or NE but increase NE release in vitro

To determine whether the stroke-induced loss of intracellular MPO and the enhanced levels of MPO and NE in sera could be mediated by catecholamines, acetylcholine or dexamethasone, we determined the effect of each of the compounds in vitro. Healthy donors’ whole venous blood samples were incubated with each of the compounds for 24 h. These treatments did not alter intracellular MPO (see Additional file 3A) or NE content (see Additional file 3B) in granulocytes or monocytes.

In supernatants, there was a small but statistically significant increase in NE concentration following treatment with 1 × 10^−7^ mol/l adrenaline (*p* < 0.0001), which was not confirmed at the higher concentrations of 1 x 10^-5^ mol/l adrenaline. Acetylcholine induced NE release at the highest concentration tested at 5.5 × 10^−4^ mol/l (*p* = 0.0259). MPO release was not induced under any of the conditions investigated (see Additional file 4).

### Catecholamines and dexamethasone decreased NET release in vitro, acetylcholine had an opposite effect

**Fig. 2:**
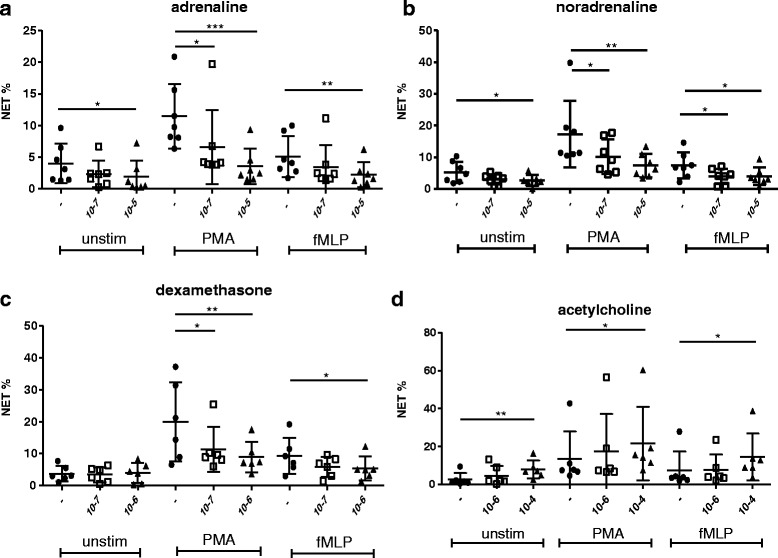
NET percentage after incubation with adrenaline, noradrenaline, dexamethasone and acetylcholine. Effect of in vitro administration of 1 × 10^−7^ M and 1 × 10^−5^ M adrenaline (**a**) and noradrenaline (**b**); 2.5 × 10^−7^ M and 2.5 × 10^−6^ M dexamethasone (**c**); and 5.5 × 10^−6^ M and 5.5 × 10^−4^ M acetylcholine (**d**) on the percentage of NET-producing cells. After incubating blood of healthy donors with hormones and acetylcholine for 4 h at room temperature, neutrophils were isolated, and NETs were induced using PMA or fMLP or left unstimulated (*n*_a,b_ = 7; *n*_c,d_ = 6). *p < 0.05; **p < 0.01; ***p < 0.005. The mean and SD ranges are given. For **a**–**c**, ANOVA and Bonferroni comparisons as post hoc tests were used. Data in **d** were assessed by Friedman test and Dunn’s multiple comparison test. Repeated measures tests were used within each of the three stimulation conditions

We reported earlier that NET area is significantly reduced post-stroke [1]. Here, we investigate the in vitro effect of hormones and neurotransmitters, which are highly increased after stroke, on NETosis; (i) adrenaline, (ii) noradrenaline, (iii) dexamethasone, a glucocorticoid substitute, and (iv) acetylcholine were tested. Neutrophil of healthy donor’s samples were isolated, and NETosis was induced in vitro by PMA and fMLP and compared to unstimulated conditions (Unstim). PMA is a strong inducer of NET, whereas fMLP has a moderate potency [19]. The percentage of NET-releasing cells in response to PMA was significantly downregulated by adrenaline (PMA_Epi_: *p* = 0.0003), noradrenaline (PMA_Norepi_: *p* = 0.0033), and dexamethasone (PMA_Dexa_: *p* = 0.0087) in a dose-dependent manner. The same tendency was observed in unstimulated and fMLP-treated samples (Unstim_Epi_: *p* = 0.0106; Unstim_Norepi_: *p* = 0.0448_;_ Unstim_Dexa_: *p* = 0.5880; fMLP_Epi_: *p* = 0.0149; fMLP_Norepi_: *p* = 0.0317; fMLP_Dexa_: *p* = 0.0376) (Fig. 2a–c). In line with these findings, the area covered by PMA-induced NETs was significantly reduced by 1 × 10^−5^ M adrenaline (Unstim_Epi_: *p* = 0.9669; PMA_Epi_: *p* = 0.0161; fMLP_Epi_: *p* = 0.0545) (see Additional file 5). Stimulation with acetylcholine induced the opposite effect. The percentage of cells that released NETs was significantly and dose-dependently increased in unstimulated and stimulated samples (Unstim_ACh_: *p* = 0.012; PMA_ACh_: *p* = 0.029; fMLP_ACh_: *p* = 0.0081) (Fig. 2d). The area covered by NETs was significantly increased under unstimulated conditions and with fMLP treatment (Unstim_Ach_: *p* = 0.0167; PMA_ACh_: *p* = 0.1741; fMLP_ACh_: *p* = 0.0431) (see Additional file 5).

### r-tPA reduces the efficiency of phagocytosis and the oxidative burst and increases NE release

Exposure to r-tPA is common in ischaemic stroke patients. Potential immunological effects of r-tPA may influence patients’ clinical courses and skew the results of immunological studies performed in stroke patients. Here, we analysed the effects of r-tPA on MPO, NE, phagocytosis, oxidative burst, NETosis and cytokine production in healthy donor’s samples in vitro.

r-tPA treatment significantly increased the in vitro release of NE into the supernatant in a dose-dependent manner but did not influence intracellular MPO or NE amounts. (NE_0 μg/ml(mean ± SD)_ 21.26 ± 2.28 ng/ml; NE_0.5 μg/ml_ 24.55 ± 3.42 ng/ml, NE_1 μg/ml_ 27.75 ± 3.75 ng/ml; *p* < 0.0001). In addition, MPO release was not altered (see Additional file 6).

**Fig. 3:**
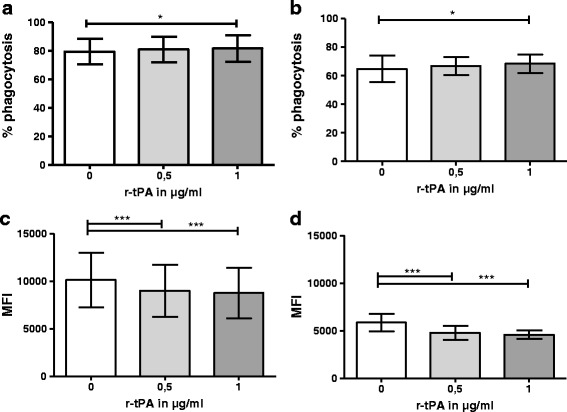
Influence of r-tPA on phagocytosis. PBMCs of healthy donors were incubated with 0.5 or 1 μg/ml r-tPA for 4 h at 37 °C in 5% CO_2_. Phagocytosis was then induced using FITC-labelled opsonized *E. coli*. The percentage of phagocytosing cells (**a, b**) and the efficacy of phagocytosis defined as the MFI (**c, d**) were investigated for granulocytes (**a, c**) and monocytes (**b, d**) (*n* = 10). *p < 0.01; ***p < 0.005. The mean and SD ranges are given. Repeated measures ANOVA and Bonferroni comparisons as post hoc tests were used

**Fig. 4:**
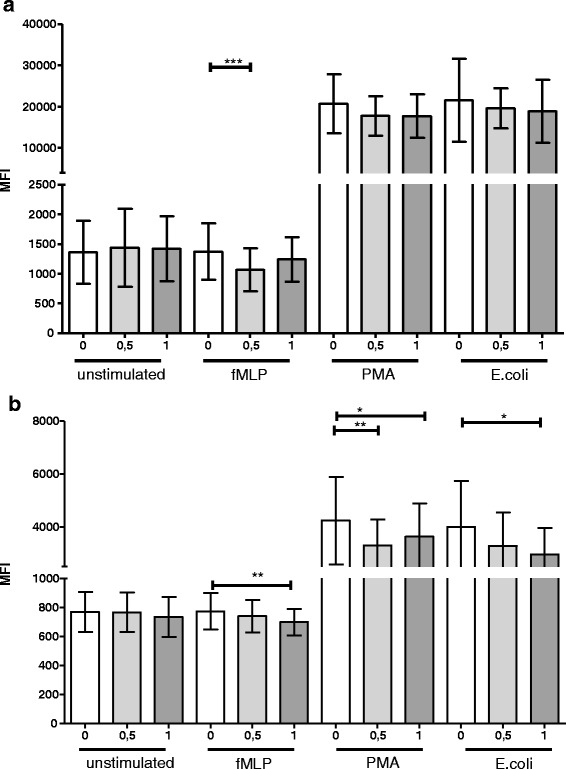
Influence of r-tPA on oxidative burst. Oxidative burst analysis was performed using blood from healthy donors for granulocytes (**a**) and monocytes (**b**). Blood samples of healthy donors were incubated with 0.5 or 1 μg/ml r-tPA for 4 h at 37 °C in 5% CO_2_. The oxidative burst was then induced using fMLP or PMA, and the efficacy (defined as the mean fluorescence intensity, MFI) of ROS was measured by flow cytometry (*n* = 10). *p < 0.05; **p < 0.01; ***p < 0.005. The mean and SD ranges are given. Repeated measures ANOVA and Bonferroni comparisons as post hoc tests were used within each of the four stimulation conditions

Whereas the percentage of reacting phagocytes was only minimally affected, (granulocytes: phagocytosis_0 μg/ml(mean ± SD)_79.52 ± 8.99%; phagocytosis_0.5 μg/ml(mean ± SD)_ 81.09 ± 8.87%; phagocytosis_1 μg/ml(mean ± SD)_ 81.90 ± 9.31%; *p* = 0.0422; monocytes: phagocytosis_0 μg/ml (mean ± SD)_ 64.88 ± 9.37%; phagocytosis_0.5 μg/ml(mean ± SD)_ 66.93 ± 6.21%; phagocytosis_1 μg/ml(mean ± SD)_ 68.47 ± 6.48%; *p* = 0.0185) (Fig. 3a, b), high- and low-r-tPA concentrations significantly decreased the numbers of phagocytosed *E. coli* per monocyte or granulocyte (granulocytes: MFI_0 μg/ml(mean ± SD)_ 10123 ± 2874; MFI_0.5 μg/ml(mean ± SD)_ 8990 ± 2734; MFI_1 μg/ml(mean ± SD)_ 8751 ± 2671; *p* < 0.0001; monocytes: MFI_0 μg/ml (mean ± SD)_ 5863 ± 927.8; MFI_0.5 μg/ml(mean ± SD)_ 4766 ± 742.0; MFI_1 μg/ml(mean ± SD)_ 4577 ± 454.3; *p* < 0.0001) (Fig. 3c, d).

r-tPA did not alter the percentages of unstimulated, fMLP-treated, PMA-treated or *E. coli*-treated cells that underwent oxidative burst, although general increases in the percentages of cells that underwent bursts due to fMLP, PMA and *E. coli* stimuli were observed (data not shown). However, there were less radicals per granulocyte in fMLP-stimulated cells treated with 0.5 μg/ml r-tPA (fMLP MFI_0 μg/ml_: _(mean ± SD)_ 1376 ± 471.9; fMLP MFI_0.5 μg/ml_ 1067 ± 360.0; fMLP MFI_1 μg/ml_ 1244 ± 373.9; *p* = 0.0004) even though fMLP did not increase the amount of radicals per cell over unstimulated levels (Fig. 4a). Using more powerful stimuli, PMA and *E. coli*, a similar tendency was observed but did not reach statistical significance for granulocytes. In monocytes, all three stimulants reached statistical significance (Fig. 4b) (fMLP MFI_0 μg/ml_: _(mean ± SD)_ 773.5 ± 126.1; fMLP MFI_0.5 μg/ml_ 740 ± 113; fMLP MFI_1 μg/ml_ 699 ± 90.23; *p* = 0.0086; PMA MFI_0 μg/ml_: _(mean ± SD)_ 4236 ± 1659; PMA MFI_0.5 μg/ml_ 3291 ± 979; PMA MFI_1 μg/ml_ 3625 ± 1250; *p* = 0.0033; *E. coli* MFI_0 μg/ml_: _(mean ± SD)_ 4000 ± 1725; *E. coli* MFI_0.5 μg/ml_ 3283 ± 1261; *E. coli* MFI_1 μg/ml_ 2952 ± 998.3; *p* = 0.0427).

The percentages of NET-releasing cells and the area covered by NETs remained unchanged by preincubating with r-tPA (data not shown). In addition, no significant changes in cytokine secretion were measured in PHA- or anti-CD3/anti-CD28-stimulated PBMCs incubated with r-tPA for 72 h (see Additional file 7A + B).

## Discussion

Stroke induces immune alterations such as suppression of antibacterial defences by the innate immune system. As previously published, two key antibacterial defence steps, the oxidative burst and NETosis, are impaired in stroke patients [1]. Furthermore, the molecular mechanisms linking this functional loss to cerebral ischaemia remain unknown. Here, we investigated the intra- and extracellular levels of two key enzymes, MPO and NE, that are involved in NETosis and the oxidative burst post-stroke.

In stroke patients, intracellular MPO was reduced in granulocytes, and a similar finding was also observed in monocytes. MPO is the key enzyme involved in free radical generation during oxidative burst, and the enzyme is also involved in chromatin decondensation, a key step in NETosis. Thus, the reduction of intracellular MPO could contribute to the impaired oxidative burst as well as the reduced NET area in stroke patients.

Although the intracellular amount of MPO was reduced, which may result in impaired intracellular killing of engulfed bacteria, the extracellular levels of MPO and NE were elevated in stroke patients. This result is in line with an earlier report [20]. The increased amounts of MPO and NE post-stroke might be caused by increased release due to phagocyte cell death including NETosis by degradation of NET and post-stroke granulocytosis [21]. It is known that secreted NE plays a role in the regulation of local inflammatory immune responses via the proinflammatory cytokine degradation, MMP-9 activation, TGF-β generation and tissue remodelling [22, 23]. Substrates of NE include fibrin, fibronectin and coagulation factors [24]. NE also promotes microvascular injury of the lungs [25]. However, the impact of extracellular MPO and NE on post-stroke immune responses and patient outcomes still needs to be determined. In addition, sample size within our study has to be regarded as limitation.

To identify the factors that trigger MPO and NE modulation in stroke, we investigated the role of catecholamines, dexamethasone and acetylcholine. None of these factors altered the amounts of intracellular MPO or NE, although adrenaline and acetylcholine increased NE release into the supernatant. This mechanism could contribute to the elevated levels of NE in patient sera. Moreover, catecholamines and dexamethasone decreased NETosis while acetylcholine was a stimulator. Catecholamines mediate their effects through adrenergic receptors which include α_1_, α_2_ and β subtypes [26]. Glucocorticoids bind to the cytoplasmatic glucocorticoid receptor [27] and acetylcholine to nicotinic acetylcholine receptors [28] and muscarinic acetylcholine receptors [29]. It is known that hormone concentrations also span a wide range in patients within the first 24 h post-stroke [5] and that hormone effects can vary depending on receptor subtype, hormone concentration and receptor expression. Future experiments should be investigating combined hormone or selective receptor effects.

Many stroke patients are exposed to r-tPA, which is a common recanalization therapy for cerebral ischaemia. We observed significant r-tPA-induced inhibition of phagocytosed bacteria and radical production per cell in vitro, whereas we detected no r-tPA effect on the pattern of cytokines released by lymphocytes.

It is difficult to assess r-tPA effects in vivo, as patients who receive thrombolysis are by definition distinct from patients who do not receive this treatment. Although the clinical relevance of this finding remains to be determined, r-tPA treatment may be an unrecognised confounder in studies designed to determine stroke-induced immune alterations. In addition to neurotoxicity rt-PA exerts within the brain it might have beneficial effects in regard to neutrophil function by reducing the ability of the cells to produce free radicals within this compartment.

## Conclusions

Reduced intracellular MPO could explain the impaired oxidative burst and reduced NET area in stroke patients. This mechanism should be targeted in future studies. The novel finding that r-tPA impairs bactericidal defences in vitro raises the question as to whether this treatment may increase the risk of developing a post-stroke infection. Patients treated successfully with r-tPA were not reported to be at a higher risk for infection. The beneficial effects of timely recanalization clearly outweigh potential off-target effects of r-tPA in this patient group. However, this outcome may be different among patients who undergo failed recanalization, who should therefore be closely monitored for infection or even receive preventive treatment. A clinical study should be carried out to test this hypothesis, and existing data from studies of stroke patients should be retrospectively subanalysed to determine the effects of r-tPA.

## Additional files

Additional file 1: Netosis in an adult control. In vitro imaging of NETs forming neutrophils with representative images for one control subject. A brightfield image allowed quantification of total cells, SYTOX® Green allowed quantification of NETosis based on size of events and comparison of different stimuli. One representative example of the unstimulated and one PMA stimulated sample is shown. Fiji Software was used to measure NET area. Images of SYTOX® Green stained cells were taken with a Leica DMI 4000 B microscope; exposure time = 170 ms. (PDF 2057 kb)

Additional file 2: Monocytic intracellular expression (MFI) of NE and MPO per cell in stroke patients versus controls. Stroke patients were analysed and compared to healthy controls. MPO (A) and NE (B) were measured intracellularly in monocytes using flow cytometry and are expressed in MFI. MPO was measured on the day of stroke admission (d0) and on days 1, 3 and 5 after stroke (d1, d3, d5) (A; n_crtl_ = 14, n_d0_ = 14, n_d1_ = 18, n_d3_ = 14, n_d5_ = 16). NE was measured on day 1 (d1) after stroke onset, day 3 (d3) and day 5 (d5) by flow cytometry (B, n_crtl_ = 10, n_d1_ = 10, n_d3_ = 10, n_d5_ = 10), and the mean and SD ranges are given. ANOVA was used for the data in A and B. (PDF 2057 kb)

Additional file 3: Influence of adrenaline, noradrenaline, dexamethasone and acetylcholine on intracellular NE and MPO in vitro. A: Intracellular MPO of granulocytes and monocytes of healthy donors treated with adrenaline, noradrenaline, dexamethasone or acetylcholine (n = 10). The mean and SD ranges are given. B: Intracellular NE of granulocytes and monocytes treated with adrenaline, noradrenaline, dexamethasone or acetylcholine (n = 10). The mean and SD ranges are given. (PDF 2057 kb)

Additional file 4: Influence of adrenaline, noradrenaline, dexamethasone and acetylcholine on NE and MPO release in vitro. NE and MPO-ELISA of supernatants of PBMCs (of healthy donors) treated with 1 × 10^−7^ M (low) or 1 × 10^−5^ M (high) adrenaline and noradrenaline; 2.5 × 10^−7^ M (low) or 2.5 × 10^−6^ M (high) dexamethasone; and 5.5 × 10^−6^ M (low) or 5.5 × 10^−4^ M (high) acetylcholine. If the data failed a Gaussian distribution test by the D‘agostino & Pearson omnibus normality test, the data were analysed by the Friedman test for repeated measures and Dunn’s post hoc test. *p < 0.05; **p < 0.01. The medians are given (n = 10). (PDF 2057 kb)

Additional file 5: Influence of adrenaline, noradrenaline, dexamethasone and acetylcholine on mean NET area. Effect of in vitro administration of 1 × 10^−7^ M or 1 × 10^−5^ M adrenaline (A) and noradrenaline (B); 2.5 × 10^−7^ M or 2.5 × 10^−6^ M dexamethasone (C); and 5.5 × 10^−6^ M or 5.5 × 10^−4^ M acetylcholine (D) on NET area was tested. After incubation of blood of healthy donors with hormones and acetylcholine for 4 h at room temperature, neutrophils were isolated, and NETs were induced using PMA or fMLP or left unstimulated (n_A,B_ = 7; n_C,D_ = 6). *p < 0.05. The mean and SD ranges are given. ANOVA and Bonferroni comparisons as post hoc tests were used. Repeated measures tests were used within each of the three stimulation conditions. (PDF 2057 kb)

Additional file 6: Influence of r-tPA on MPO and NE. PBMCs of healthy donors were incubated with 0.5 or 1 μg/ml r-tPA for 4 h at 37 °C in 5% CO_2_. Afterwards, MPO and NE were measured intracellularly in granulocytes by flow cytometry (A, C), expressed in MFI, and in the supernatant using ELISA (B, D), expressed in ng/ml (n = 10). *p < 0.01; ***p < 0.005. Mean and SD ranges are given. Repeated measures ANOVA and Bonferroni comparisons as post hoc tests were used. (PDF 2057 kb)

Additional file 7: A: Influence of r-tPA on cytokine production with PHA stimulation. Production of INF-γ, IL-2, -4, -5, -6, -9, -13, -17A, -17 F, -21, -22 and TNF-α in the supernatants of PBMCs after 72 h of incubation with r-tPA. PBMCs were stimulated with PHA to induce cytokine production. IL-10 was not induced in any of the conditions and is not shown. As an example, the data from PBMCs isolated from venous blood of healthy donors anticoagulated with EDTA are shown. The results for heparin-anticoagulated venous blood were similar and are not shown. (n = 4). The means are given. B: Influence of r-tPA on cytokine production with anti-CD3/anti-CD28 stimulation. Production of INF-γ, IL-2, -10, -5, -6, -9, -13, -17A, -17 F, -21, -22 and TNF-α in supernatants of PBMC after 72 h of incubation with r-tPA. PBMCs of healthy donors were stimulated with anti-CD3/anti-CD28 to induce cytokine production. IL-4 was not induced in any of the conditions and is not shown. As an example, the data from PBMCs isolated from venous blood anticoagulated with EDTA are shown. The results for heparin-anticoagulated venous blood were similar and are not shown (n = 4). The means are given. (PDF 2057 kb)

## Abbreviations

Ach: Acetylcholine
APC: Allophycocyanin
Dexa: Dexamethasone
DNA: Deoxyribonucleic acid
*E. coli*: *Escherichia coli*
EDTA: Ethylenediaminetetraacetic acid
ELISA: Enzyme-linked immunosorbent assay
Epi: Adrenaline
FITC: Fluorescein isothiocyanate
fMLP: *N*-formylmethionine-leucyl-phenylalanine
HEPES: 4-(2-hydroxyethyl)-1-piperazineethanesulfonic acid
MFI: Mean fluorescence intensity
MPO: Myeloperoxidase
NE: Neutrophil elastase
NETs: Neutrophil extracellular traps
NIHSS: National Institute of Health Stroke Scale Score
Norepi: Noradrenaline
PBMC: Peripheral blood mononuclear cells
PE: Phycoerythrin
PMA: Phorbol 12-myristate 13-acetate
ROS: Reactive oxygen species
r-tPA: Recombinant tissue plasminogen activator
Unstim/US: Unstimulated conditions
