# Supplementary figures and images for: Ischaemic stroke and the recanalization drug tissue plasminogen activator interfere with antibacterial phagocyte function
Source: J Neuroinflammation. 2017 Jul 21;14:140. doi: 10.1186/s12974-017-0914-6 (PMC5521108; doi:10.1186/s12974-017-0914-6)

brightfield

Sytox stained

unstimulated

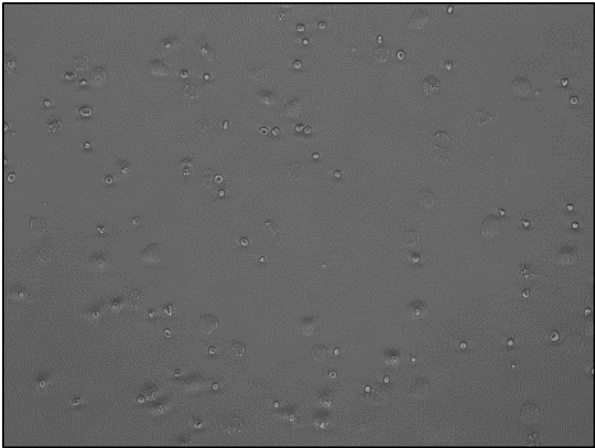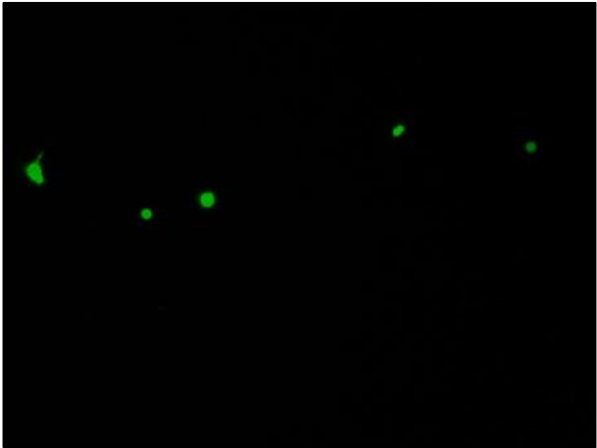

PMA stimulated

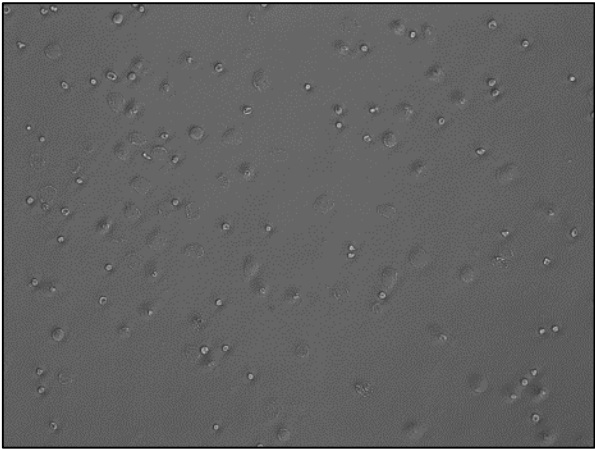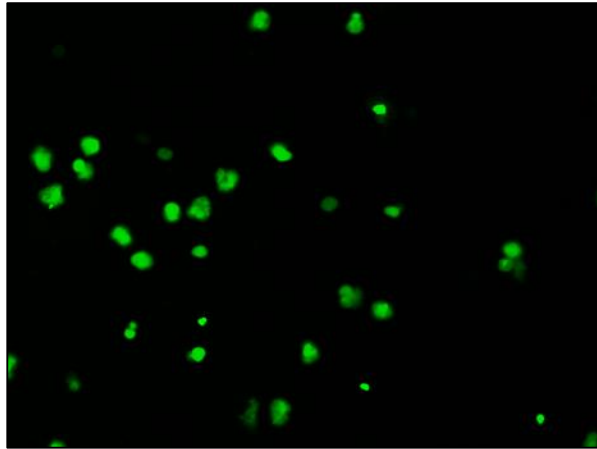

Supplement: Supplementary file 1 — Netosis in an adult control. In vitro imaging of NETs forming neutrophils with representative images for one control subject. A brightfield image allowed quantification of total cells, SYTOX® Green allowed quantification of NETosis based on size of events and comparison of different stimuli. One representative example of the unstimulated and one PMA stimulated sample is shown. Fiji Software was used to measure NET area. Images of SYTOX® Green stained cells were taken with a Leica DMI 4000 B microscope; exposure time = 170 ms. (PDF 2057 kb) [file 12974_2017_914_MOESM1_ESM.pdf]

A

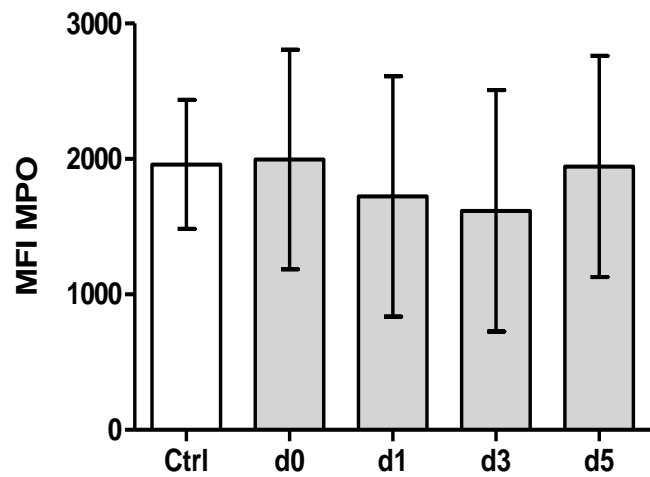

B

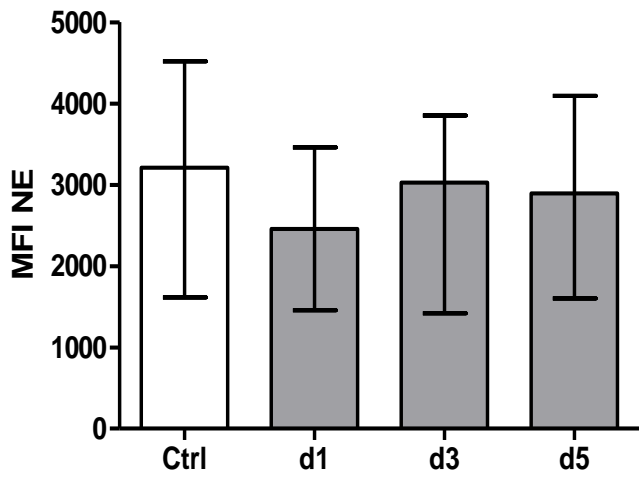

Supplement: Supplementary file 2 — Monocytic intracellular expression (MFI) of NE and MPO per cell in stroke patients versus controls. Stroke patients were analysed and compared to healthy controls. MPO (A) and NE (B) were measured intracellularly in monocytes using flow cytometry and are expressed in MFI. MPO was measured on the day of stroke admission (d0) and on days 1, 3 and 5 after stroke (d1, d3, d5) (A; ncrtl = 14, nd0 = 14, nd1 = 18, nd3 = 14, nd5 = 16). NE was measured on day 1 (d1) after stroke onset, day 3 (d3) and day 5 (d5) by flow cytometry (B, ncrtl = 10, nd1 = 10, nd3 = 10, nd5 = 10), and the mean and SD ranges are given. ANOVA was used for the data in A and B. (PDF 2057 kb) [file 12974_2017_914_MOESM2_ESM.pdf]

A

Intracellular MPO

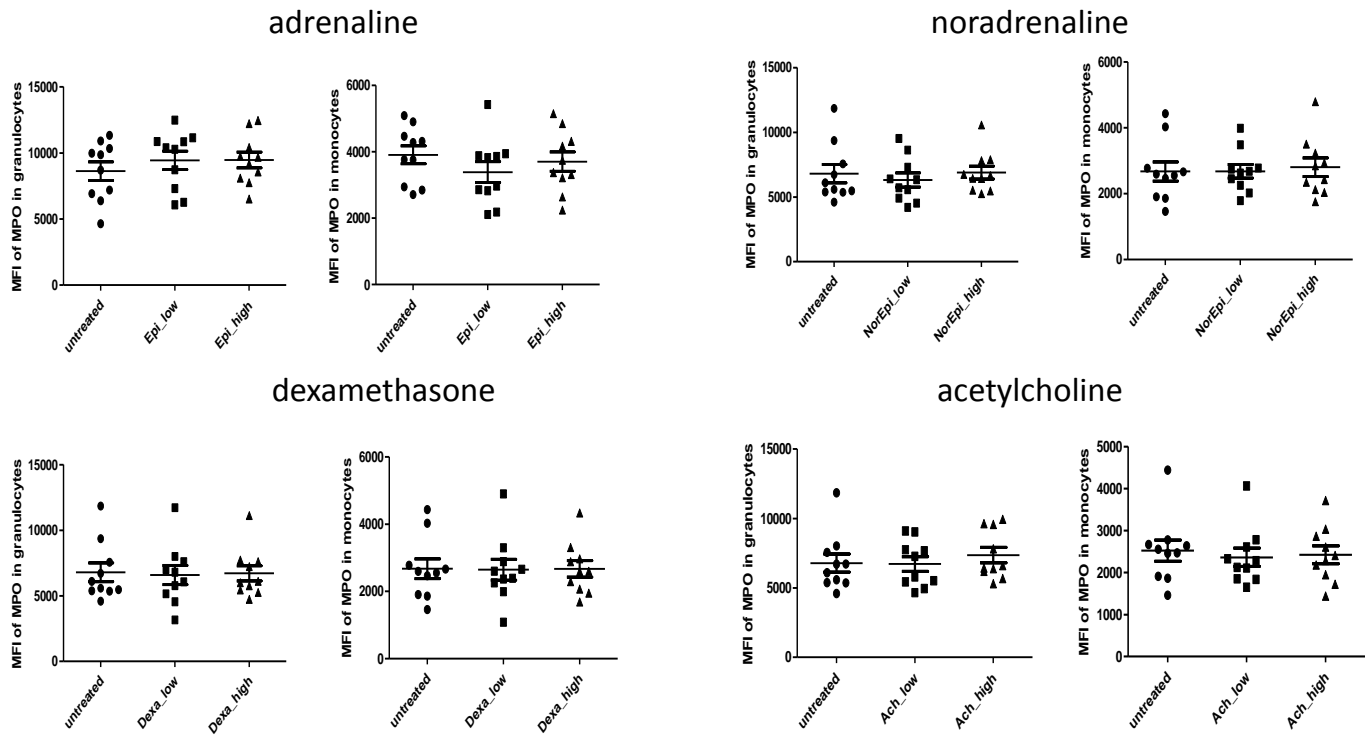

B

Intracellular NE

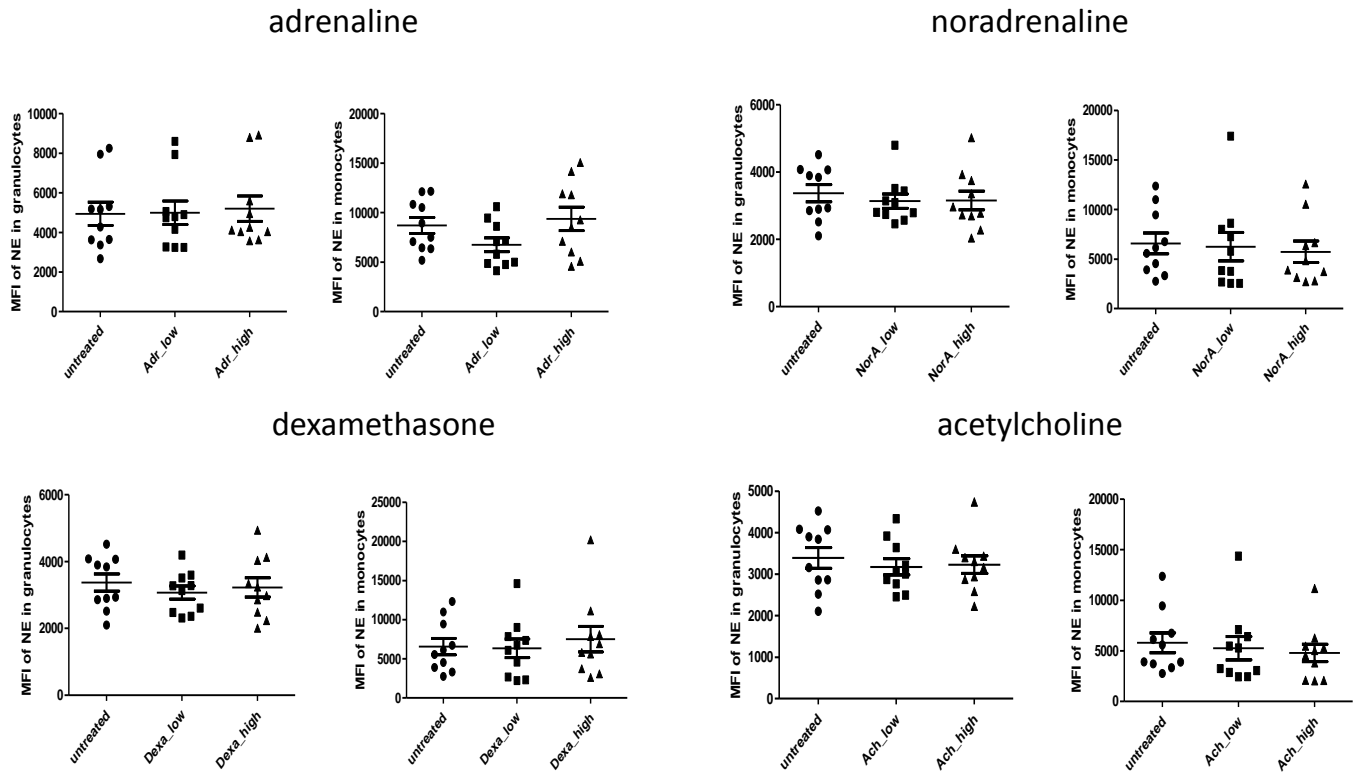

Supplement: Supplementary file 3 — Influence of adrenaline, noradrenaline, dexamethasone and acetylcholine on intracellular NE and MPO in vitro. A: Intracellular MPO of granulocytes and monocytes of healthy donors treated with adrenaline, noradrenaline, dexamethasone or acetylcholine (n = 10). The mean and SD ranges are given. B: Intracellular NE of granulocytes and monocytes treated with adrenaline, noradrenaline, dexamethasone or acetylcholine (n = 10). The mean and SD ranges are given. (PDF 2057 kb) [file 12974_2017_914_MOESM3_ESM.pdf]

NE

MPO

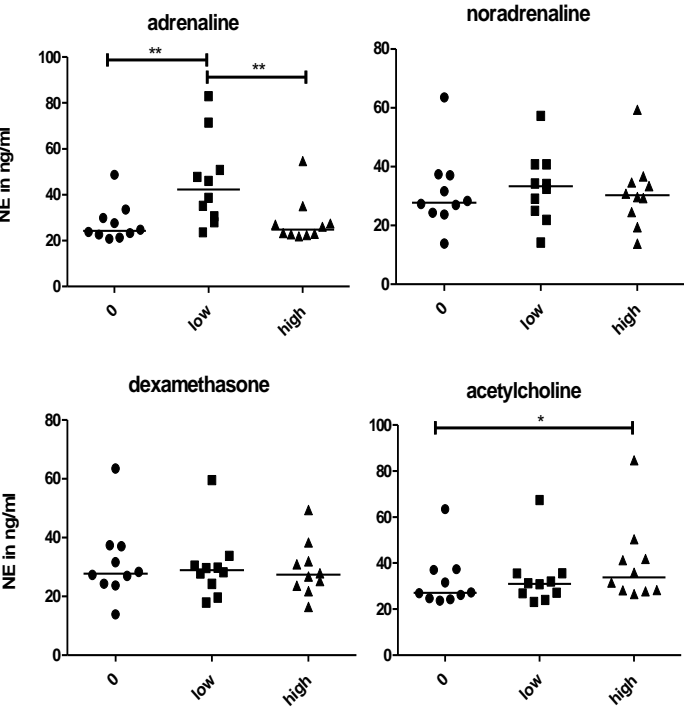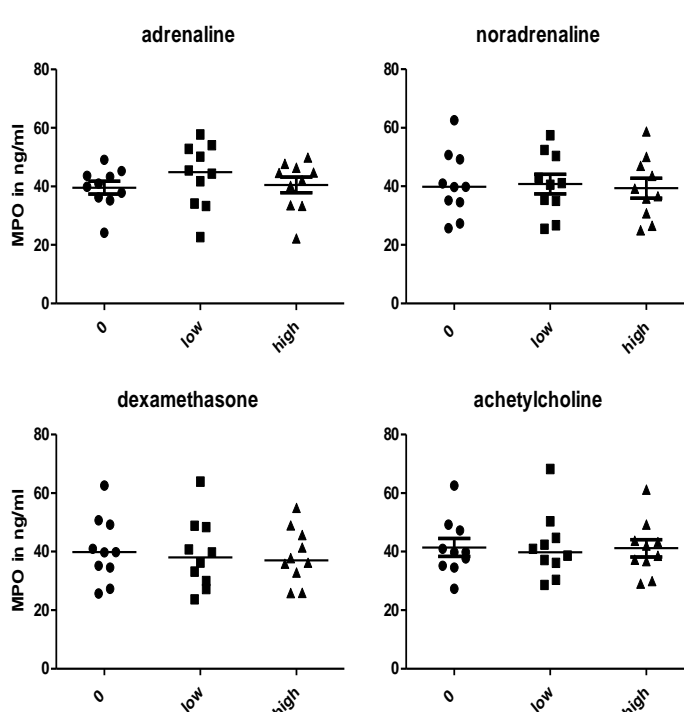

Supplement: Supplementary file 4 — Influence of adrenaline, noradrenaline, dexamethasone and acetylcholine on NE and MPO release in vitro. NE and MPO-ELISA of supernatants of PBMCs (of healthy donors) treated with 1 × 10^−7^ M (low) or 1 × 10^−5^ M (high) adrenaline and noradrenaline; 2.5 × 10^−7^ M (low) or 2.5 × 10^−6^ M (high) dexamethasone; and 5.5 × 10^−6^ M (low) or 5.5 × 10^−4^ M (high) acetylcholine. If the data failed a Gaussian distribution test by the D‘agostino & Pearson omnibus normality test, the data were analysed by the Friedman test for repeated measures and Dunn’s post hoc test. *p < 0.05; **p < 0.01. The medians are given (n = 10). (PDF 2057 kb) [file 12974_2017_914_MOESM4_ESM.pdf]

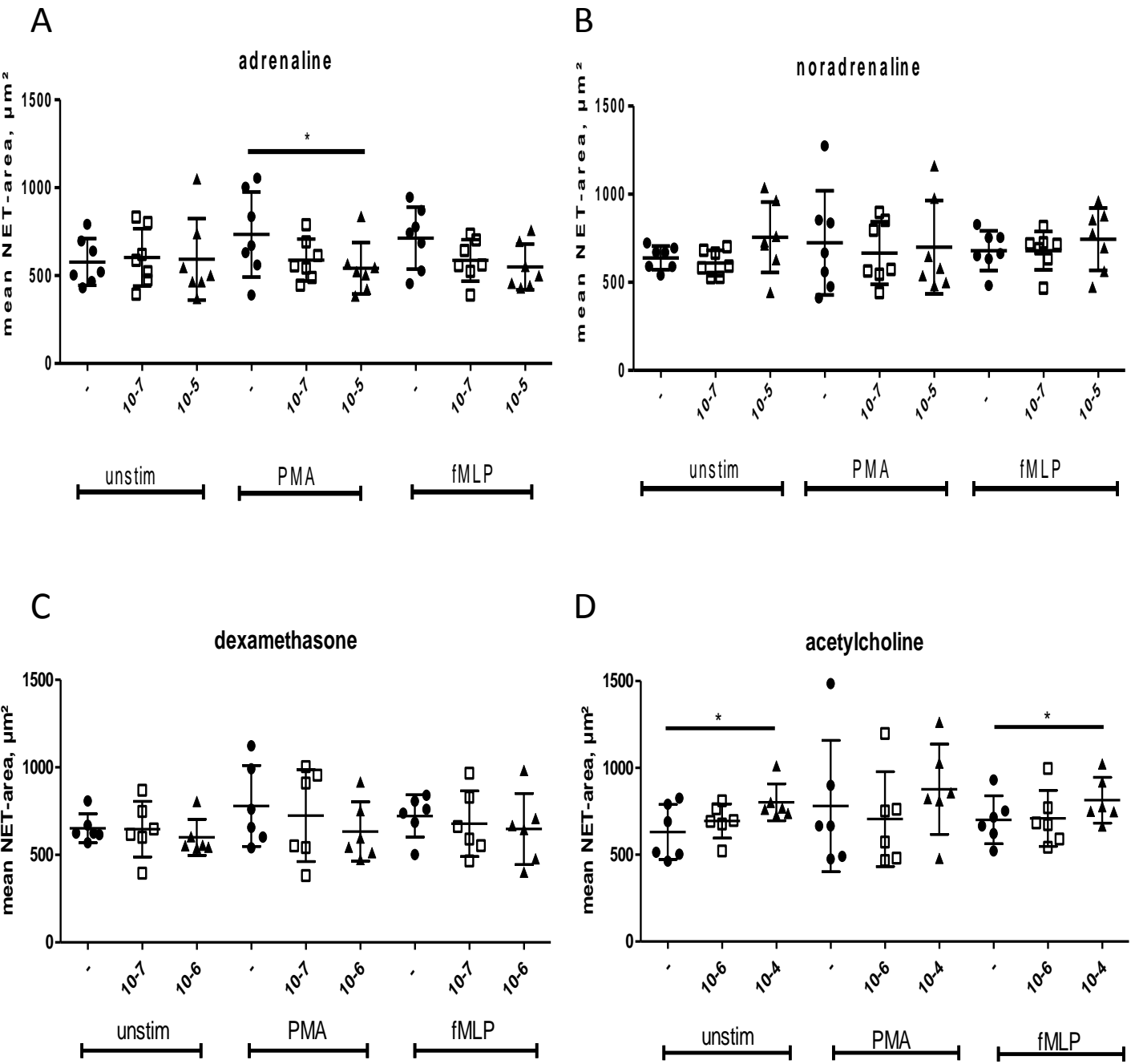

Supplement: Supplementary file 5 — Influence of adrenaline, noradrenaline, dexamethasone and acetylcholine on mean NET area. Effect of in vitro administration of 1 × 10^−7^ M or 1 × 10^−5^ M adrenaline (A) and noradrenaline (B); 2.5 × 10^−7^ M or 2.5 × 10^−6^ M dexamethasone (C); and 5.5 × 10^−6^ M or 5.5 × 10^−4^ M acetylcholine (D) on NET area was tested. After incubation of blood of healthy donors with hormones and acetylcholine for 4 h at room temperature, neutrophils were isolated, and NETs were induced using PMA or fMLP or left unstimulated (nA,B = 7; nC,D = 6). *p < 0.05. The mean and SD ranges are given. ANOVA and Bonferroni comparisons as post hoc tests were used. Repeated measures tests were used within each of the three stimulation conditions. (PDF 2057 kb) [file 12974_2017_914_MOESM5_ESM.pdf]

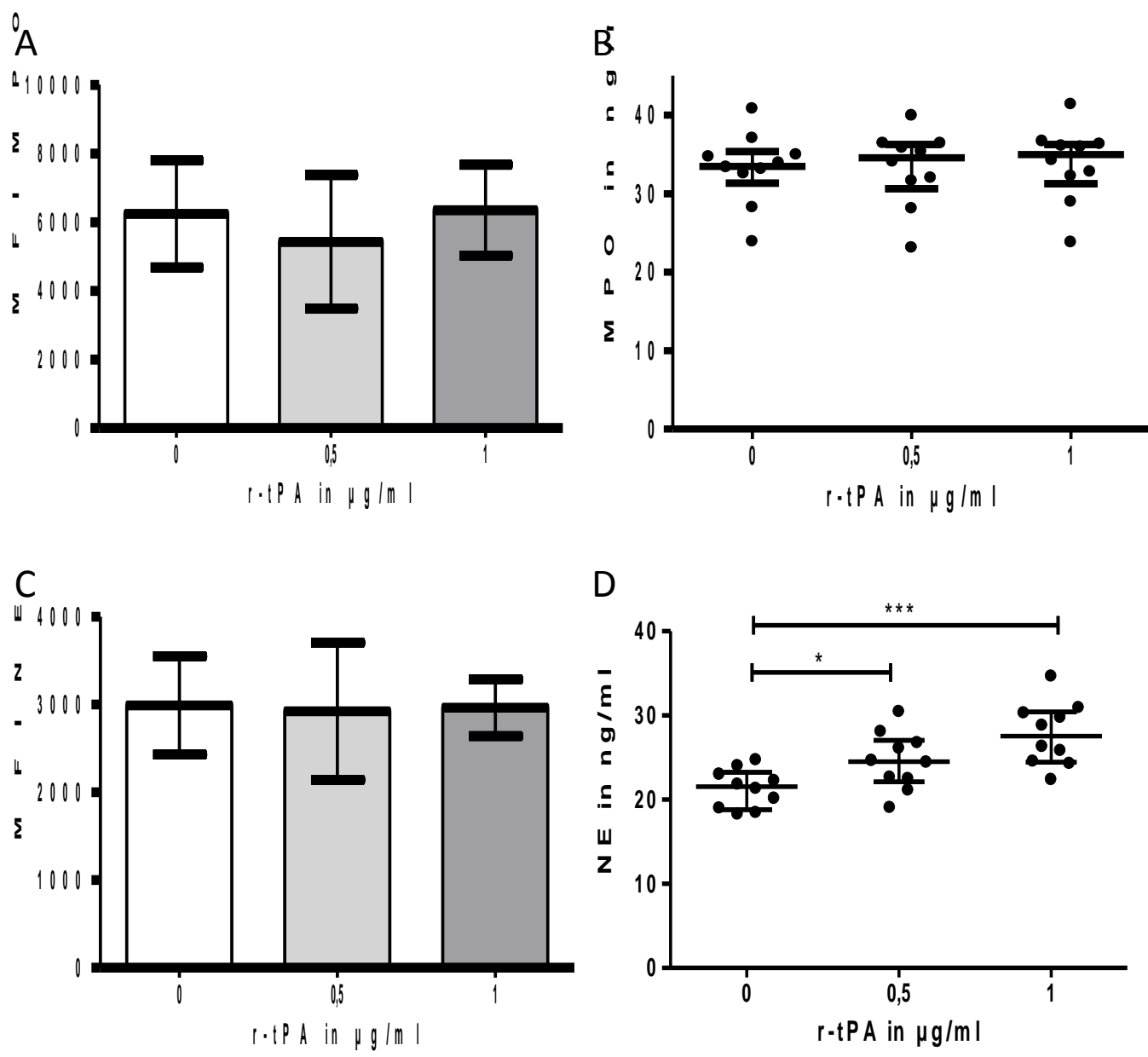

Supplement: Supplementary file 6 — Influence of r-tPA on MPO and NE. PBMCs of healthy donors were incubated with 0.5 or 1 μg/ml r-tPA for 4 h at 37 °C in 5% CO2. Afterwards, MPO and NE were measured intracellularly in granulocytes by flow cytometry (A, C), expressed in MFI, and in the supernatant using ELISA (B, D), expressed in ng/ml (n = 10). *p < 0.01; ***p < 0.005. Mean and SD ranges are given. Repeated measures ANOVA and Bonferroni comparisons as post hoc tests were used. (PDF 2057 kb) [file 12974_2017_914_MOESM6_ESM.pdf]

Additional file VII A

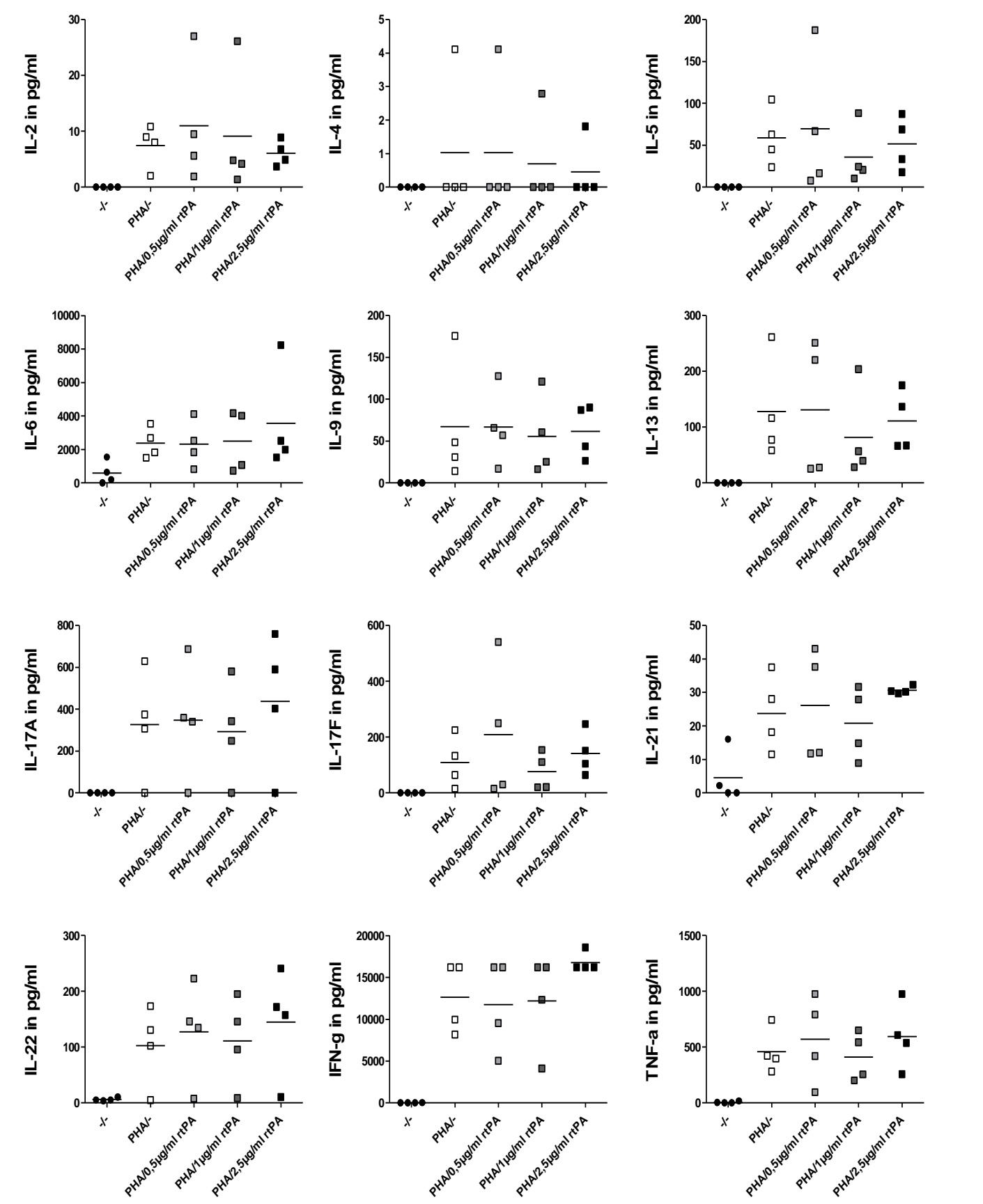

Additional file VII B

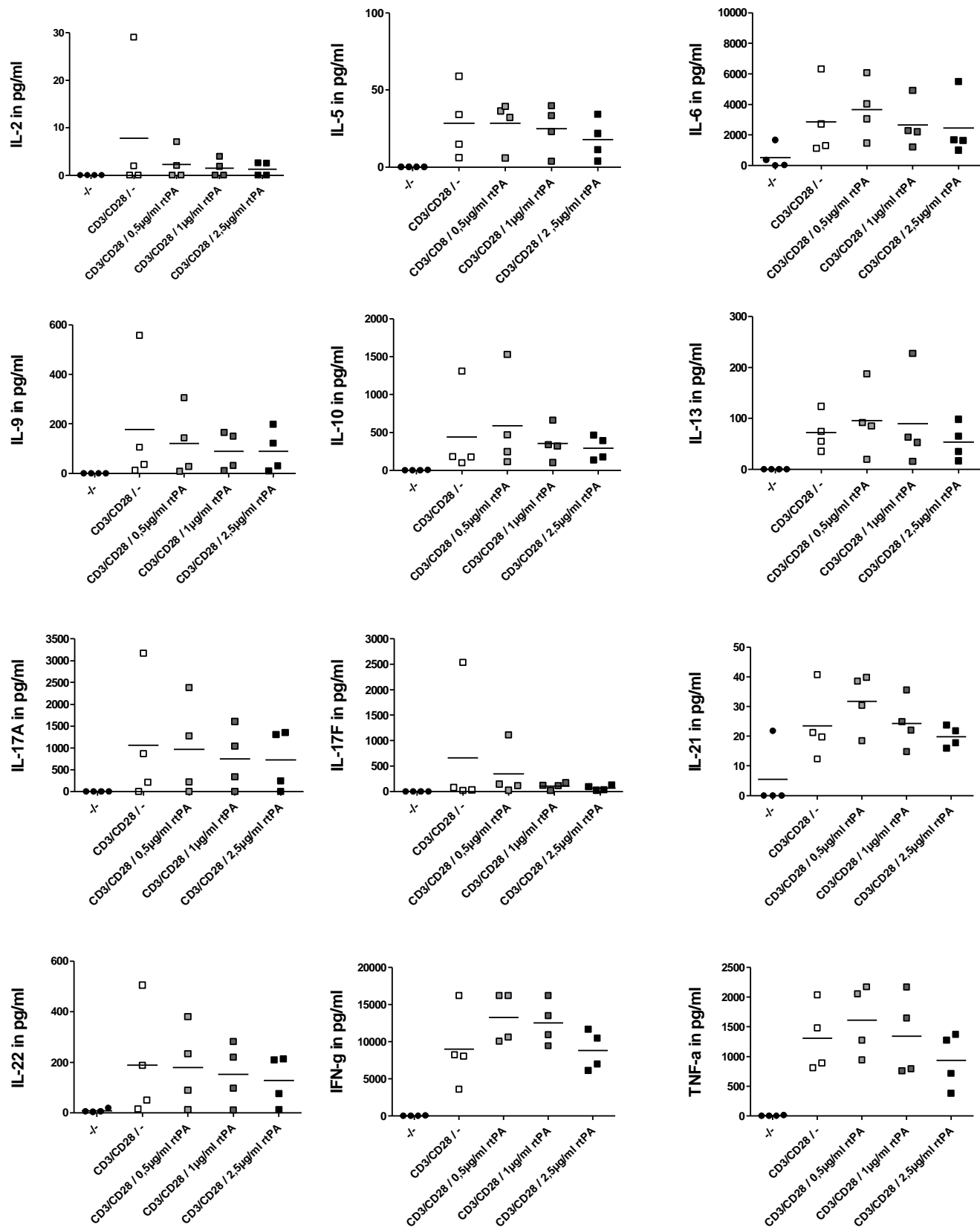

Supplement: Supplementary file 7 — A: Influence of r-tPA on cytokine production with PHA stimulation. Production of INF-γ, IL-2, -4, -5, -6, -9, -13, -17A, -17 F, -21, -22 and TNF-α in the supernatants of PBMCs after 72 h of incubation with r-tPA. PBMCs were stimulated with PHA to induce cytokine production. IL-10 was not induced in any of the conditions and is not shown. As an example, the data from PBMCs isolated from venous blood of healthy donors anticoagulated with EDTA are shown. The results for heparin-anticoagulated venous blood were similar and are not shown. (n = 4). The means are given. B: Influence of r-tPA on cytokine production with anti-CD3/anti-CD28 stimulation. Production of INF-γ, IL-2, -10, -5, -6, -9, -13, -17A, -17 F, -21, -22 and TNF-α in supernatants of PBMC after 72 h of incubation with r-tPA. PBMCs of healthy donors were stimulated with anti-CD3/anti-CD28 to induce cytokine production. IL-4 was not induced in any of the conditions and is not shown. As an example, the data from PBMCs isolated from venous blood anticoagulated with EDTA are shown. The results for heparin-anticoagulated venous blood were similar and are not shown (n = 4). The means are given. (PDF 2057 kb) [file 12974_2017_914_MOESM7_ESM.pdf]
